# Environmental DNA metabarcoding revealed the impacts of anthropogenic activities on phytoplankton diversity in Dianchi Lake and its three inflow rivers

**DOI:** 10.1002/ece3.10088

**Published:** 2023-05-19

**Authors:** Yuanyuan Lin, Wenjun Zhong, Xiaowei Zhang, Xiaohua Zhou, Liwei He, Jiacheng Lv, Zheng Zhao

**Affiliations:** ^1^ Academician Workstation for Ecological Health Assessment and Rehabilitation of Rivers and Lakes in Kunming, Key Laboratory of River and Lake Ecological Health Assessment and Restoration in Yunnan Province, Kunming Dianchi Lake Environmental Protection Collaborative Research Center Kunming University Kunming China; ^2^ State Key Laboratory of Pollution Control and Resource Reuse, School of the Environment Nanjing University Nanjing China

**Keywords:** Dianchi Lake, environmental DNA metabarcoding, environmental variables, inflow rivers, phytoplankton community

## Abstract

Phytoplankton diversity is closely related to environmental variables and has been widely used in ecological health assessment of rivers and lakes. Combining advantages of DNA‐based identification and high‐throughput sequencing technology, environmental DNA (eDNA) metabarcoding permits a new measurement for biodiversity monitoring in aquatic ecosystems. However, it had rarely been used to explore the variability and similarity of phytoplankton diversity between lake and its inflow rivers and the effects of environmental variables on phytoplankton. This study utilized eDNA metabarcoding to investigate the spatial distribution of phytoplankton and the impacts of environmental variables on the phytoplankton diversity in Dianchi Lake (one of the most polluted urban lakes in China) and its main inflow rivers (Panlong River, Baoxiang River, and Chai River). A total of 243 distinct phytoplankton taxa were detected, covering 9 phyla, 30 classes, 84 orders, and 132 families, and the taxonomic richness of rivers was higher than that of Dianchi Lake. Distinct biodiversity patterns (e.g., community structure, dominant taxon, ɑ‐diversity) were exhibited among Dianchi Lake and its three inflow rivers, but similar biodiversity patterns were also observed in Dianchi Lake and the estuarine sites. The patterns of phytoplankton diversity were closely related to environmental variables, which were associated with pollution sources from different anthropogenic activities (e.g., urbanization, water diversion, industrial and agricultural activities). The primary environmental variables correlated with phytoplankton diversity varied in different habitats. The total phosphorus (TP) and chemical oxygen demand (COD) positively correlated with the phytoplankton community structures in Dianchi Lake, whereas negatively correlated in Panlong River and Baoxiang River. The total nitrogen (TN) positively correlated with the phytoplankton community structures in Baoxiang River and Chai River but negatively correlated in Dianchi Lake. Overall, this study provides insights on the phytoplankton diversity monitoring and the conservation of its diversity and healthy management of Dianchi Lake.

## Graphical abstract

This study investigated the distribution characteristics of phytoplankton at Dianchi Lake and its three inflow rivers through environmental DNA metabarcoding technology, the distribution characteristics reflected the effects of anthropogenic activities on phytoplankton diversity (e.g., community structure, unique dominant taxon, alpha diversity). It provides sights on environmental response mechanisms of phytoplankton community, and the health assessment and restoration of Dianchi Lake.

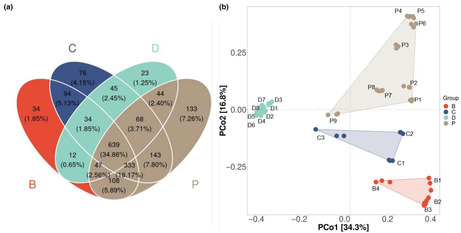

## 1 INTRODUCTION

Due to pervasive anthropogenic activities (e.g., population expansion, industrialization, and urbanization), biodiversity and ecosystem functions have been subjected to worldwide decline in freshwater ecosystems especially in urban lakes and rivers (Cardinale et al., 2012; Li et al., 2022; Reid et al., 2019). The monitoring and protection of aquatic biodiversity have attracted more and more attention from the public and government. Dianchi Lake is an urban lake located in Kunming, Yunnan province, China, which plays important roles in urban development of Kunming. However, with increasing anthropogenic disturbances (e.g., increasing domestic and industrial sewage, excessive fertilizer use), Dianchi Lake has become one of the most polluted lakes in China (Li, Janssen, et al., 2019; Yang et al., 2018), eutrophication especially large harmful algal blooms occurred frequently and severely in recent years (Huang et al., 2014; Mu et al., 2019; Wang et al., 2019). The protection and management of Dianchi Lake has become increasingly important and urgent.

Phytoplankton, consisting of prokaryotes (Cyanobacteria) and multiple eukaryotic algae, play vital roles in nutrient recycling and energy flow and are recognized as important components of lakes and rivers (Reynolds et al., 2006). Phytoplankton rapidly respond to fluctuating water environmental variables such as temperature, pH, dissolved oxygen, and nutrient salts, and which can lead to the changes in community structure of entire aquatic organisms from phytoplankton to fish directly or indirectly through the carbon cycle, nutrient cycle, and food web (De Senerpont Domis et al., 2013; Reynolds et al., 2006). Phytoplankton are recommended as environmental indicator organisms for the variation of aquatic ecosystems induced by anthropogenic activities (Aura et al., 2020; Birk et al., 2012; Phillips et al., 2013). Lakes and rivers are two different ecosystems with distinct physical and chemical characteristics; therefore, the phytoplankton communities were individual and reflected the ecological status of the water bodies (Soballe & Kimmel, 1987). Research has shown that eutrophication, particularly excess dissolved inorganic nitrogen (DIN) and increasing nitrogen (N)/phosphorus (P), could be responsible for the shift in the phytoplankton community structure and the bloom of harmful algal in the Changjiang (Yangtze) River estuary (Li, Tang, et al., 2014). Based on the succession of phytoplankton community and indicator species, Nankabirwa et al. (2019) deemed that the fresh Ugandan crater lakes, which subjected to increasing anthropogenic perturbations, comprise four trophic levels and need governance and protection. Generally, the main source of lake water is from its inflow rivers, the nutrients loadings of lakes are usually positively correlated with the nutrients inputs from the inflow rivers (Bridgeman et al., 2012; Gao et al., 2021). The water quality status of lake water body is closely related to the inflow rivers, so does the phytoplankton diversity (Bridgeman et al., 2012; Dai et al., 2022). It is essential to explore the discrepancy of phytoplankton diversity between lake and its inflow rivers and the effects of environmental variables on phytoplankton to provide references for assessment and protection of lakes and rivers. However, little research on the comparison of phytoplankton community between lake and its inflow rivers and the relations to environmental variables has been done.

Traditionally, phytoplankton were monitored by their morphological characteristics, which have many defects such as time‐consuming, high cost, low species resolution, and highly dependent on the experience of appraisers (Phan et al., 2016; Reynolds et al., 2006). Difference from traditional methods, environmental DNA (eDNA) metabarcoding combined DNA‐based identification and high‐throughput sequencing technology to interpret the community structures of different habitats rapidly and accurately using their DNA directly extracted from soil, water, or air samples (Deiner et al., 2017). Recently, eDNA metabarcoding has been applied in discriminating species composition at different habitats, the early detection of invasive species and rare species etc., and suggests a new measurement for biodiversity monitoring in aquatic ecosystems, particularly in microbial groups (Deiner et al., 2017; Hering et al., 2018; Lin & Zhao, 2021; Rey et al., 2019). Analyzed the ballast water from seven ships arriving in Gijon port (south Bay of Biscay, Spain) by eDNA metabarcoding, Ardura et al. (2020) catalogued the exotic and harmful algae species in Bay of Biscay. By analyzing historical eDNA from a sediment core, Ibrahim et al. (2021) revealed two major break points of changes in the phytoplankton community that date back some 110 years, and these strongly correlated with the change in concentration of phosphorus. eDNA metabarcoding has been considered a valuable tool for phytoplankton diversity monitoring. However, it was rarely applied in Dianchi Basin, particularly in assessing the responses of phytoplankton diversity to the variation of environmental variables caused by anthropogenic activities. Only Zhang et al. (2021) explored the precision of eDNA metabarcoding technology for eukaryotic phytoplankton monitoring in Dianchi and Fuxian Lakes. Therefore, there is an urge to explore the phytoplankton diversity and improve the understanding of environmental responses of phytoplankton diversity from anthropogenic activities' impacts throughout eDNA metabarcoding method in the Dianchi Basin.

Based on eDNA metabarcoding, this study aimed to analyze the distribution characterization of phytoplankton and the correlation between environmental variables and phytoplankton community structure in Dianchi Lake and its primary inflow rivers (Panlong River, Baoxiang River, and Chai River). We test whether it had significant differences in phytoplankton diversity between Dianchi Lake and its primary inflow rivers, and which environmental variables could take charge of these differences in phytoplankton diversity such as the variations of dominant taxon relative abundances and community structure? This study provides baseline information for the ecological health assessment and restoration of Dianchi Lake, Panlong River, Baoxiang River and Chai River.

## 2 METHODS

### 2.1 Study area and sampling

**FIGURE 1:**
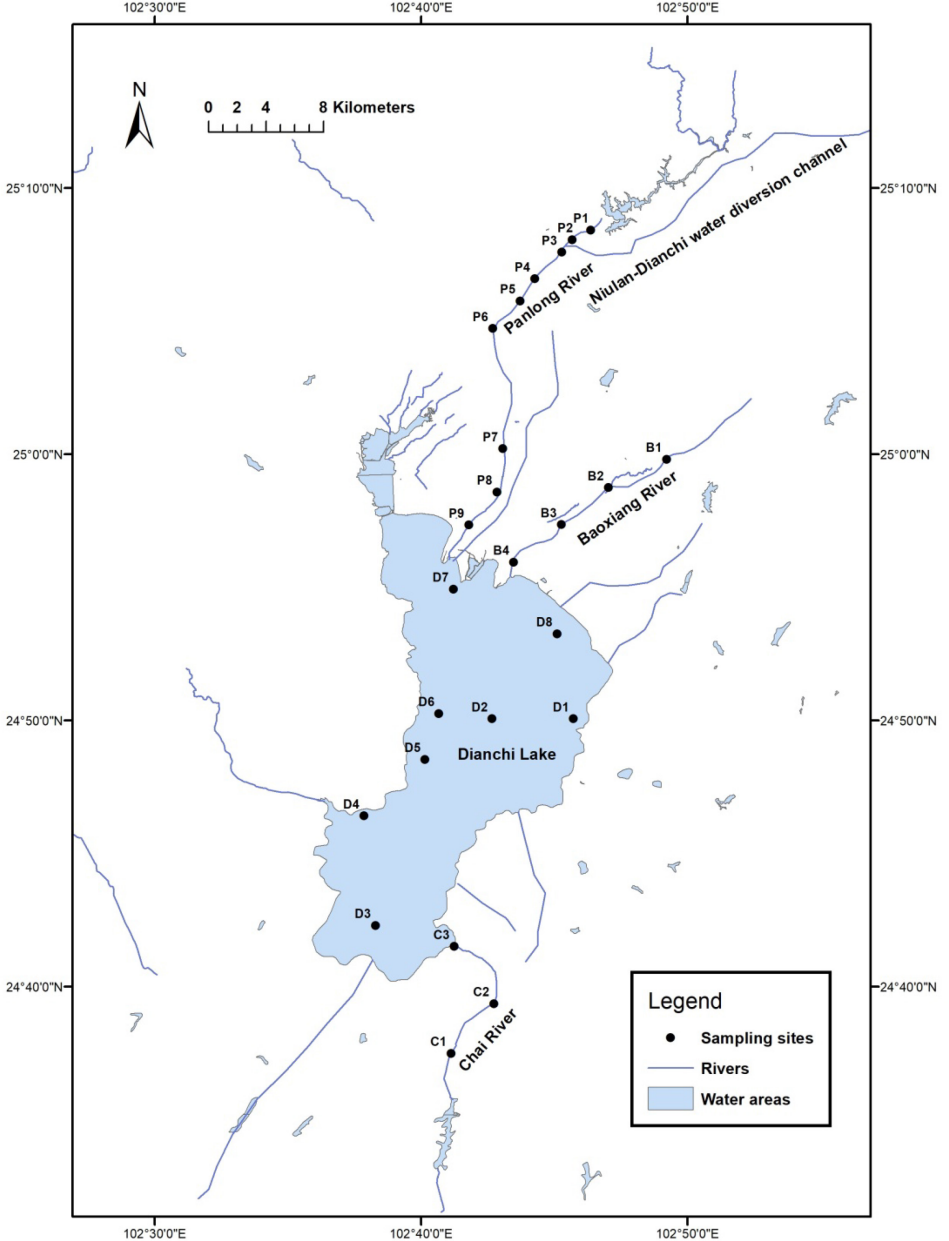
Map of the study area with sampling stations indicated. A total of 24 stations were set up, including eight sites in Dianchi Lake (D1–D8), nine sites in Panlong River (P1–P9), four sites in Baoxiang River (B1–B4), and three sites (C1–C3) in Chai River.

Dianchi Lake is an urban lake located in the middle of the Yunnan‐Guizhou Plateau, and as it is located in downstream of Kunming city, anthropogenic activities substantially impact the water quality status. In recent years, the water quality of Dianchi Lake has been deteriorating, and eutrophication resulted in the frequent occurrence of large destructive algal blooms (Huang et al., 2014; Mu et al., 2019; Wang et al., 2019). Studies showed that the inflow rivers, which were flooded with a large amount of agricultural, industrial, and urban effluents, were taken charge of the serious water pollution of Dianchi Lake primarily and made the process of managing much more difficult (Jin et al., 2017; Zhang, 2016). The management of Dianchi Lake should be based on the management of the rivers particularly on the primary inflow rivers (He et al., 2020; Xu et al., 2016). Panlong River, Baoxiang River, Chai River flow through mountainous, villages, towns, and main urban areas of Kunming into Dianchi Lake, and the impacts of anthropogenic activities on the rivers increased from upstream to downstream. Panlong River primarily suffers from urban pollution, Baoxiang River from agricultural and urban pollution, and Chai River from agricultural and phosphate ore pollution (Li, Yang, et al., 2014; Zhang, 2016). Dianchi Lake and the three inflow rivers were the investigation areas in this study. Figure 1 shows the geographic distribution of the monitoring sites, including eight sites in Dianchi Lake (D1–D8), nine sites in Panlong River (P1–P9), four sites in Baoxiang River (B1–B4), and three sites (C1–C3) in Chai River. In October 2020, 2 L of surface water was collected at each monitoring site, a volume of 1 L of water sample (Rey et al., 2019) was filtered through a 0.45 μm hydrophilic nylon membrane (MilliporeSigma) and stored at −20°C until DNA extraction, and the other liter of water was used to measure environmental variables. There were three biological replicates per site and the water samples were stored at 4°C before filtration and measuring environmental variables. The sampling controls were processed with ddH_2_O.

### 2.2 Analysis of environmental variables

Physicochemical characteristics including water temperature (WT) °C, pH, dissolved oxygen (DO) mg/L, ammonia nitrogen (${\mathrm{NH}}_{4}^{+}$) mg/L, total nitrogen (TN) mg/L, chemical oxygen demand (COD) mg/L, total phosphorus (TP) mg/L, and conductivity (C) μs/cm were measured for each sampling site. The WT, pH, and DO were measured in situ using a YSI water quality analyzer (YSI Inc.). In accordance with standard protocols (China, Ministry of Water Resources of the People‘s Republic of China, 1994), Knott's spectrophotometry, digestion‐ultraviolet spectrophotometry, potassium dichromate method, ammonium molybdate spectrophotometry, and conductivity meter method were carried out in the laboratory to analyze ${\mathrm{NH}}_{4}^{+}$, TN, COD, TP, and C, respectively.

### 2.3 DNA extraction, PCR amplification, and sequencing

According to the manufacturer's instructions, eDNA from the filter membrane was extracted using the DNeasy PowerWater Kit (Qiagen). Using the diluted eDNA samples as templates, 16S rDNA V3 hypervariable region (Amplicon size ~180 bp) targeting cyanobacteria and the 18S rDNA V9 hypervariable region (Amplicon size ~130 bp) targeting eukaryotic phytoplankton were amplified respectively. The V3 primer sequences were 341F: 5′‐ACCTACGGGRSGCWGCAG‐3′; 518 R: 5′‐GGTDTTACCGCGGCKGCTG‐3′ (Klindworth et al., 2013). The V9 primer sequences were 1380F: 5′‐TCCCTGCCHTTTGTACAC‐3′; 1510R: 5′‐CCTTCYGCAGGTTCACCTAC‐3′ (Langsley et al., 2009). Unique 12‐bp nucleotide fragments were added to the 5′‐ends of the forward primers (GenScript). Three replicates of PCR per sample were carried out in a 30 μL reaction mixture, including 15 μL of 2 × Phanta enzyme mixture (Vazyme), 1 μL of each primer (10 μM), 1 μL of template, and 12 μL of nuclease‐free water. Three negative PCR controls for every PCR process were simultaneously preformed using nuclease‐free water as DNA templates. The thermocycler conditions were as follows: 98°C for 30 s, following 30 cycles of denaturing at 98°C for 15 s, annealing at 62°C and extending at 72°C for 30 s, and with a final extension at 72°C for 5 min. The PCR products were visualized on a 2% agarose gel, and the target bands were cut and purified using the E‐Z 96 Cycle Pure Kit (Omega‐Biotek). All the purified products were quantified and pooled. The sequencing libraries were constructed using the Ion Xpress Plus Fragment Library kit (Thermo Fisher Scientific). All samples were diluted to a final concentration of 100 pM and sequenced in the Ion S5™ (Thermo Fisher Scientific) following the manufacturer's protocols.

### 2.4 Bioinformatics

Following the QIIME2 pipeline, the raw data were trimmed, sorted, and distinguished by unique sample tags (Caporaso et al., 2010). Operational taxonomic units (OTUs) were clustered at cutoff value of 97% nucleotide similarity using the UPARSE pipeline (Edgar, 2013). The taxonomy annotation for each OTU of 16S and 18S was analyzed by the Greengenes database (DeSantis et al., 2006) and PR2 (release 4.12.0) databases for prokaryote and eukaryote communities, respectively. Details of these procedures have been previously described (Li et al., 2018). Since the research area was located in inland China, we referred the book “The Freshwater Algae of China, Systematics, Taxonomy and Ecology” (Hu & Wu, 2006) to screen out the phytoplankton OTUs for further analysis. And referred to the articles (Malashenkov et al., 2021; Zhang et al., 2022), OTUs at species/genera level were used to show the phytoplankton diversity.

### 2.5 Statistical analysis

The metabarcoding data were summarized in separate OTU tables; the reads of OTUs present simultaneously in at least two replicates were averaged as the actually detected reads of each OTU at one site; the OTU tables were available at Dryad (https://doi.org/10.5061/dryad.dbrv15f5s). Shannon–Wiener's diversity index was used to calculate the ɑ‐diversity of phytoplankton in each sampling site. The variation in community composition among sites was visualized using a principal coordinate analysis (PCoA) based on Bray–Curtis dissimilarity. A permutational multivariate analysis of variance (PERMANOVA) with 999 permutations was used to evaluate the community differences among habitats. Spearman's correlation analysis, Mantel test, and redundancy analysis (RDA) were used to reflect the relationship between phytoplankton community and environmental variables. *t*‐Tests were performed to show the differences of OTU abundance and ɑ‐diversity among Dianchi Lake, Panlong River, Baoxiang River, and Chai River. All these statistical analysis used the “vegan” package in R software 3.6.0 (R Core Team, 2019).

## 3 RESULTS

### 3.1 Sequencing results and phytoplankton community profile

A total of 5,797,423 high‐quality (>Q20) reads were obtained by high‐throughput sequencing of 72 samples from 24 sites in Dianchi Lake and the three inflow rivers. The number of reads per sample was 38,017 ± 8163 (mean ± SE) for the 16S‐V3 assays and 42,502 ± 15,085 (mean ± SE) for the 18S‐V9 assays. A total of 13,091 prokaryotic OTUs and 5741 eukaryotic OTUs were identified from the 2,737,266 reads (16S‐V3) and 3,060,157 reads (18S‐V9), respectively. Fewer than the 10% reads/OTUs (12,989 reads/428 OTUs for 16S‐V3 and 278,961 reads/657 OTUs for 18S‐V9) could not be assigned to datasets. The rarefaction curves saturated (Figure S1), which indicated that the sequencing data could be used for subsequent analysis.

**FIGURE 2:**
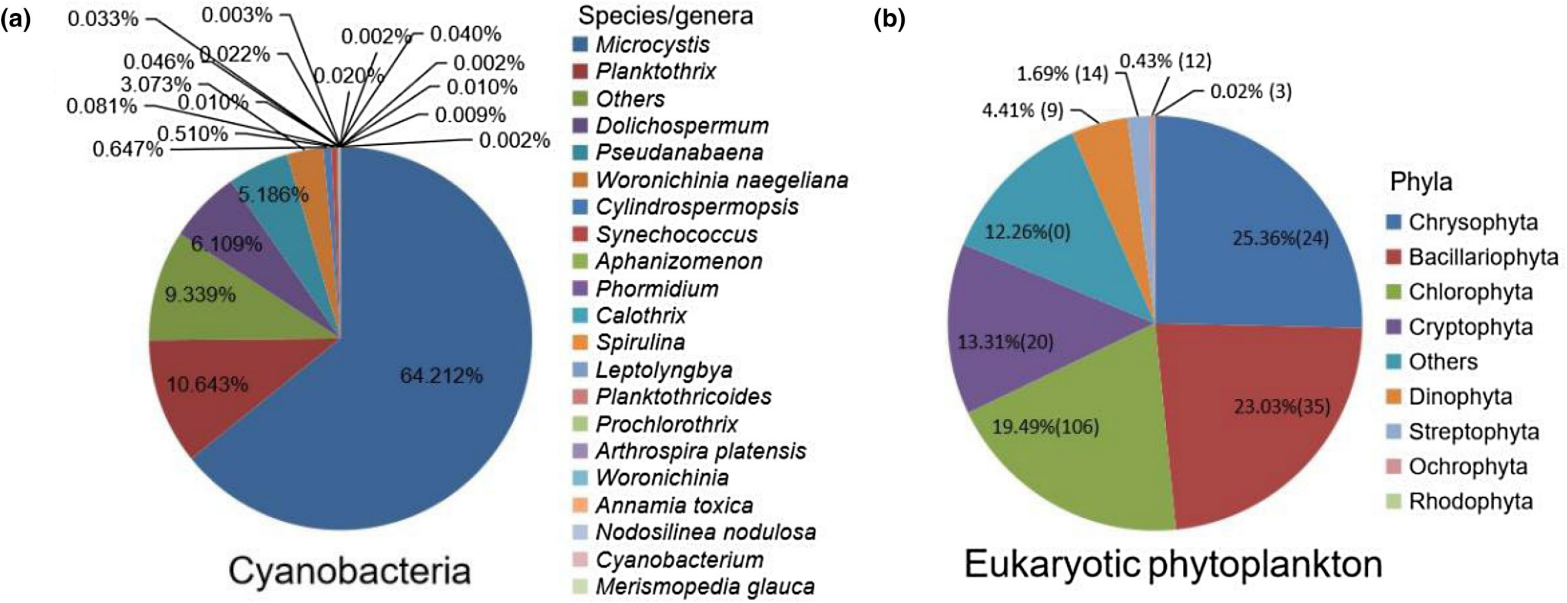
Community composition of phytoplankton in Dianchi Lake and the three inflow rivers. (a) Cyanobacteria taxa. (b) Eukaryotic phytoplankton taxa. Percentages are the relative abundance of reads. The numbers in parentheses are the number of taxa at the species/genera level.

A total of 1833 OTUs were assigned to the phytoplankton community and reserved for further analysis in this study. A total of 704 OTUs could be annotated to the genus level above, covering 9 phyla, 30 classes, 84 orders, 132 families, and 243 species/genera (182 genera and 180 species). From 266 cyanobacterial OTUs, 20 cyanobacterial species/genera were identified. The majority of cyanobacterial reads belonged to *Microcystis*, accounting for 64.21% of the total cyanobacterial reads, and *Planktothrix*, *Dolichospermum*, *Pseudanabaena*, and *Woronichinia naegeliana* were predominant in the Cyanobacteria (Relative abundance >2%) (Figure 2a). From 1567 eukaryotic phytoplankton OTUs, 223 eukaryotic phytoplankton species/genera were detected, covering 8 phyla, 27 classes, 77, orders, and 120 families. Chrysophyta, Bacillariophyta, and Chlorophyta dominated in the eukaryotic phytoplankton community, accounting for 25.36%, 23.03%, and 19.49% of the total reads, respectively (Figure 2b).

### 3.2 Spatial distribution of the phytoplankton community

**FIGURE 3:**
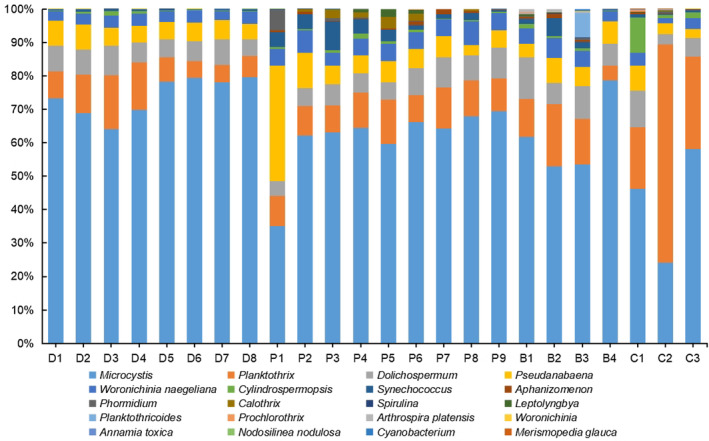
Patterns of distribution of the cyanobacterial community in different sampling sites using the relative abundances of the different taxa at species/genera level.

**FIGURE 4:**
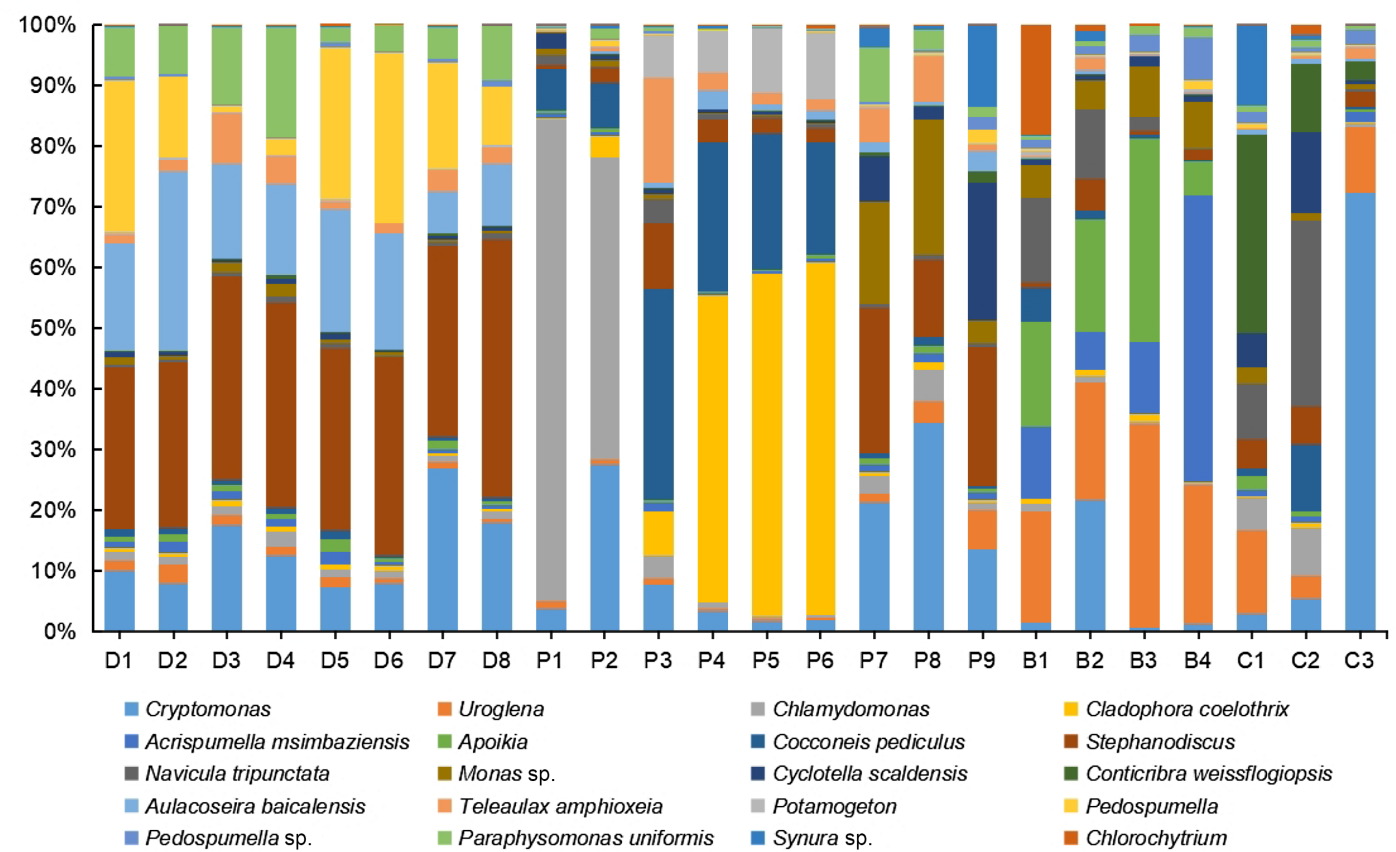
Patterns of distribution of the eukaryotic phytoplankton community in different sampling sites using the relative abundances of the top 20 species/genera.

Distinct spatial distribution patterns of phytoplankton community were observed among sampling sites (Figures 3 and 4). Although the compositions of cyanobacterial community varied among the sampling sites, *Microcystis* dominated in the cyanobacterial community, which the relative abundances were all more than 50% in each other site except in C1, C2, and P1. The compositions of eukaryotic phytoplankton community varied among the different habitats. Figure 4 showed the distribution of the top 20 species/genera taxa at each site. The patterns of distribution of these taxa in the Dianchi Lake sites (D1–D8) were relatively consistent, which *Stephanodiscus*, *Aulacoseira baicalensis*, *Pedospumella*, *Ceratium* sp., and *Cryptomonas* were accounted for most of the sequence numbers. The distribution of eukaryotic phytoplankton taxa varied among the river sampling sites. The dominant taxon in the upper regions (P1, P2) of Panlong River was *Chlamydomonas*. There was little difference in distribution patterns of the top 20 species/genera in midstream of Panlong River (P4, P5, P6), which the predominant taxa included *Cladophora coelothrix*, *Cocconeis pediculus*, and *Potamogeton* among others. The predominant taxon was *Uroglena* in the upper regions of Baoxiang River (B1), *Cryptomonas* (B2), and *Uroglena* (B3) in the middle regions, and *Acrispumella msimbaziensis* in the lower regions (B4). In the upper, middle, and lower regions of Chai River, the predominant taxa were *Conticribra weissflogiopsis*, *Navicula tripunctata*, and *Cryptomonas*, respectively.

### 3.3 Biodiversity patterns

**FIGURE 5:**
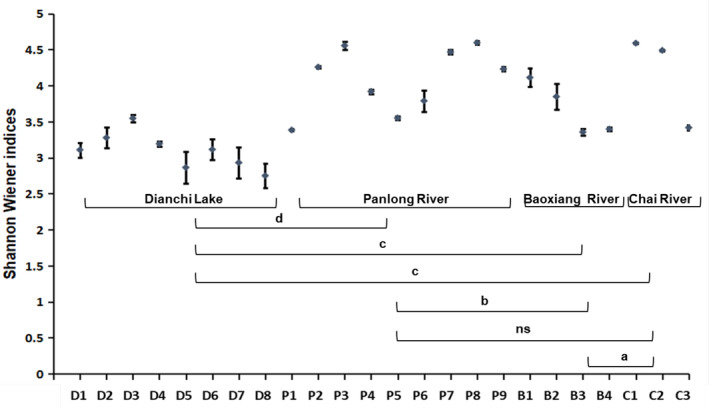
ɑ‐diversity difference at the sampling sites and different habitats. Significant terms are marked as: a, .01 < *p* < .05; b, .001 < *p* < .01; c, *p* < .001; d, *p* < .0001; ns, non‐significant, *p* > .05.

The ɑ‐diversity index reflects the diversity of the community within the sample. Clear spatial distribution patterns of phytoplankton diversity among the sampling sites were shown by the Shannon–Wiener index (Figure 5). The Shannon–Wiener indices of Dianchi Lake were between 2.74 and 3.54, which were significantly lower than those of the inflow rivers (*t*‐test, *p* < .001). The Shannon–Wiener indices in the Panlong River fluctuated greatly, with P3 and P8 peak points, whereas the Shannon–Wiener indices tended to decrease from upstream to downstream in Baoxiang River and Chai River.

**FIGURE 6:**
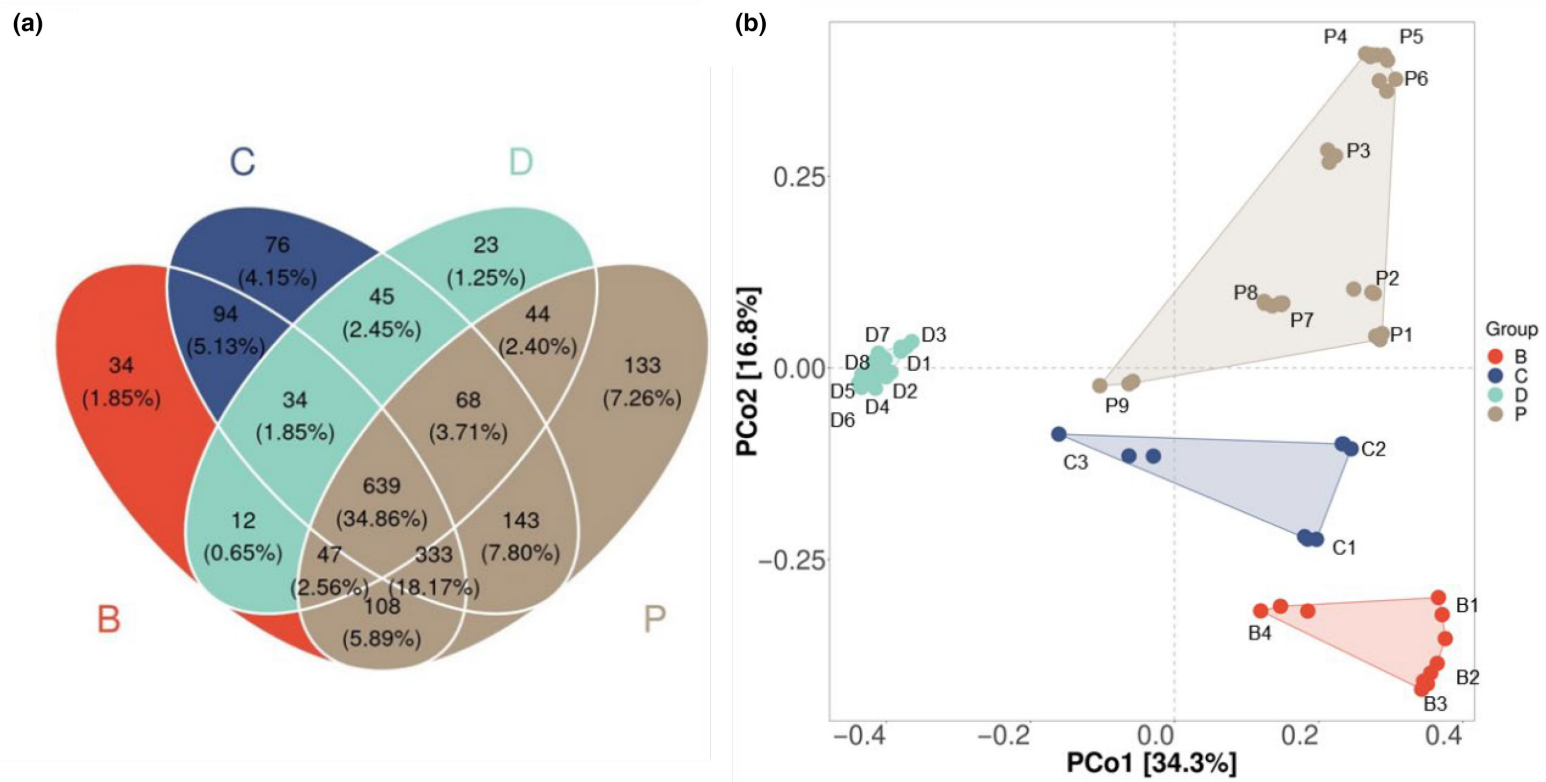
Patterns of biodiversity in Dianchi Lake and the inflow rivers. (a) Venn diagrams of OTUs distribution. (b) PCoA of phytoplankton community structure. B, C, D, and P are the abbreviations of Baoxiang River, Chai River, Dianchi Lake, and Panlong River.

Distinct biodiversity patterns were observed between the sampled habitats of Dianchi Lake and the inflow rivers. Figure 6a reflects the differences of species composition in different habitats. There were 639 common OTUs (accounting for 34.86% of the total OTU number) distributed simultaneously in Dianchi Lake and the three inflow rivers. A total of 266 unique OTUs distributed singly in a particular habitat, which the highest number of OTUs (133) was detected in Panlong River and the least (23) was in Dianchi Lake. A total of 98.75% of the OTUs distributed in Dianchi Lake were overlapped with those in inflow rivers, and the taxonomic richness of inflow rivers was much higher than that of Dianchi Lake.

PCoA (Figure 6b) and PERMANOVA tests (Table S1, *p* < .001) of phytoplankton community structure also demonstrated a clear partitioning of biota between Dianchi Lake and the inflow rivers. The samples of Dianchi Lake were clustered closely with each other, and samples from each river were also grouped together, respectively. There was no significant difference between Dianchi Lake sample sites and estuarine entry sites (B4, C3, and P9) shown by the PERMANOVA tests (Table S1, *r* ^2^ > .8879, *p* > .1), which revealed the similarity of biodiversity patterns between Dianchi Lake and the estuary sites.

### 3.4 Correlation of phytoplankton community with environmental variables

**FIGURE 7:**
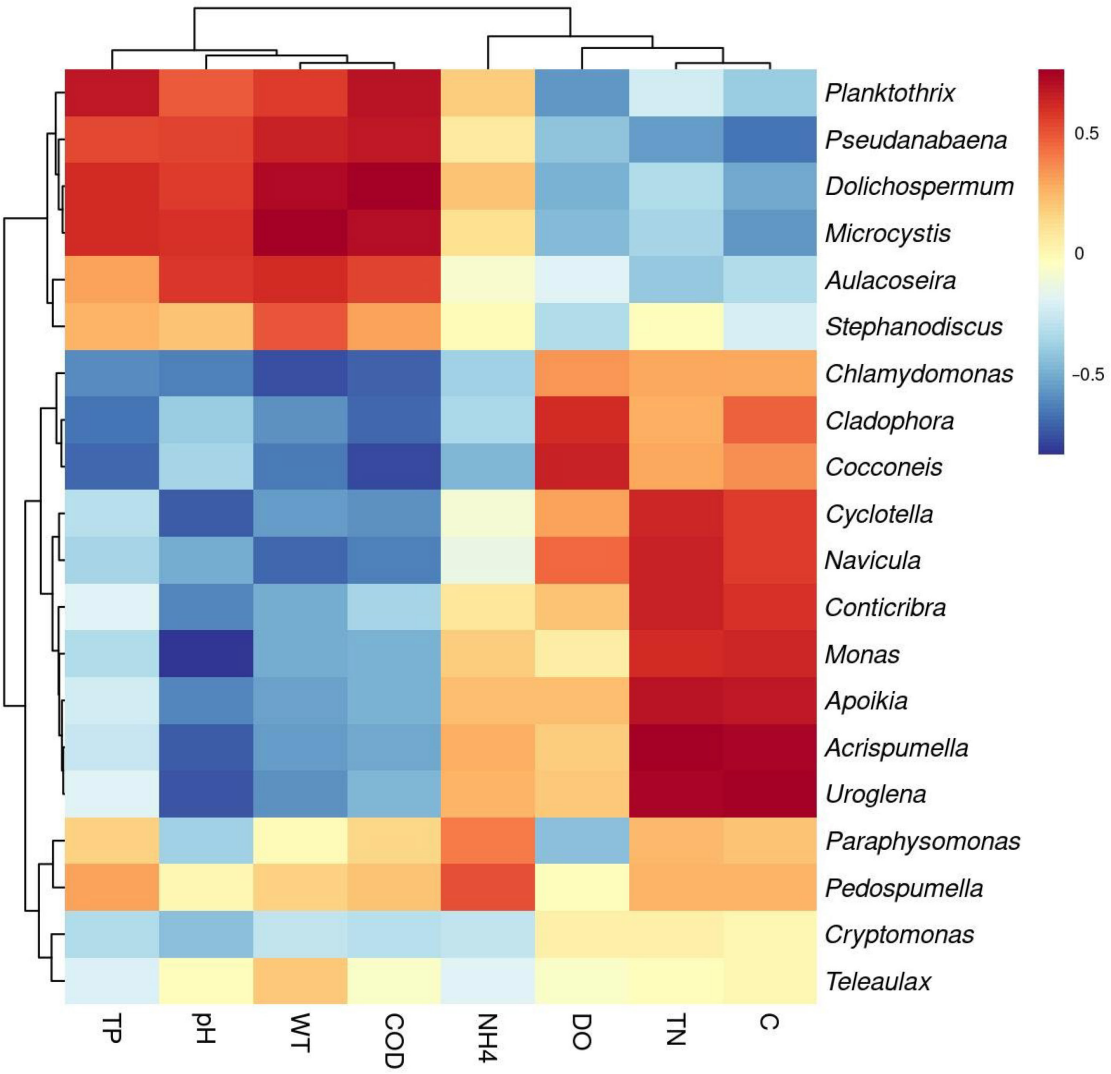
Correlation cluster analysis between environmental variables and the relative abundances of phytoplankton genera (top 20). C, conductivity; COD, chemical oxygen demand; DO, dissolved oxygen; NH_4_, ammonia nitrogen; TN, total nitrogen; TP, total phosphorus; WT, water temperature.

The impacts of environmental variables on the relative abundances of specific genera were revealed by Spearman's correlation analyses. Environmental variables were significantly related to phytoplankton abundances, Figure 7 showed the correlations between environmental variables and top 20 genera. The correlations between environmental variables and different genera were inconsistent. For example, COD and WT were positively correlated with *Microcystis* and *Dolichospermum*, TN and C were positively correlated with *Acrispumella* and *Uroglena*, whereas PH were negatively associated with *Monas*, *Acrispumella*, *Uroglena*, and *Cyclotella* (|*r*| > .7, *p* < .001, Figure 7, Table S2).

**FIGURE 8:**
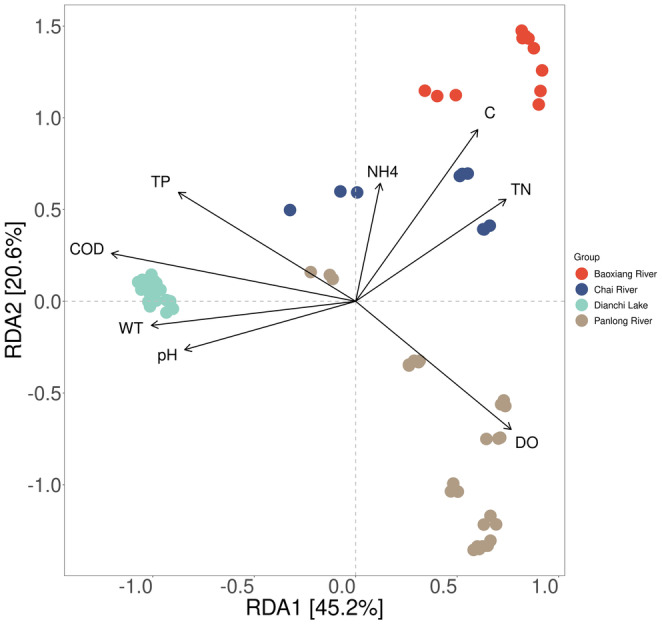
RDA ordination plot illustrating the relationships between phytoplankton community and environmental variables of Dianchi Lake and the three inflow rivers. C, conductivity; COD, chemical oxygen demand; DO, dissolved oxygen; NH_4_, ammonia nitrogen; TN, total nitrogen; TP, total phosphorus; WT, water temperature.

Correlation analysis between phytoplankton community and environmental variables was carried out to explore the key environmental stressors affecting the water quality status of Dianchi Lake and the three inflow rivers. The Mantel test results revealed that pH, WT, DO, COD, TP, TN, and C significantly affected the phytoplankton community (Table S3, Mantel's *r* < .2001, *p* < .001). A redundancy analysis demonstrated a clear partitioning of environmental stressors between Dianchi Lake and the rivers (Figure 8). COD, WT, TP, and pH were positively correlated with the phytoplankton community structures in Dianchi Lake, whereas DO, TN, and C were negatively correlated. The correlation between the phytoplankton community structures and environmental variables displayed differences among the river sampling sites. DO was the most important positive factor that affected the phytoplankton community in most of the Panlong River sites, whereas COD and TP were negative correlation factors. The phytoplankton communities in Baoxiang River and the upper and middle regions of Chai River were positively correlated with TN and C, and negatively correlated with TP, COD, WT, and pH.

## 4 DISCUSSION

### 4.1 eDNA metabarcoding revealed the composition and distribution, variability, and similarity of phytoplankton diversity in Dianchi Lake and its primary inflow rivers

eDNA metabarcoding is a promising tool for biodiversity monitoring, which is much more efficient and faster than traditional methods (Deiner et al., 2017; Rey et al., 2019). Based on eDNA metabarcoding, 243 distinct phytoplankton taxa were detected from 1833 phytoplankton OTUs in Dianchi Lake and its three inflow rivers, covering 9 phyla, 30 classes, 84 orders, and 132 families. There were 901 OTUs in Dianchi Lake, 1515 in Panlong River, 1301 in Baoxiang River, and 1432 in Chai River. The taxonomic richness of the inflow rivers was much higher than that of Dianchi Lake, and 98.75% of the OTUs in Dianchi Lake were overlapped with those in the three inflow rivers. It indicated that the richness of phytoplankton diversity in Dianchi Lake could be closely associated with the biota of these three inflow rivers. Similar results have been found in Lake Poyang and Tianmuhu, which the taxonomic diversity could be related to the biota of their inflow river network (Li, Liu, et al., 2019; Xie, Tang, et al., 2021). Anthropogenic activities such as dam building lead to hydrological disconnection, causing the decline of biodiversity, whereas water diversion could facilitate the hydrological connectivity and the radiation of aquatic organisms (Hu et al., 2021; Shao et al., 2019; Zhang et al., 2022). The taxonomic richness of Panlong River was the highest, which could be also associated with the artificial diversion of Niulan River entering the river. Dai et al. (2018) and Zhang et al. (2022) proved that water diversion from Yangtze River to Tai Lake accelerated the biotic homogenization of phytoplankton communities between connected water bodies. Similar biodiversity patterns were exhibited between Dianchi Lake and estuary sites (P9, B4, and C3) in our research, which also proved the biotic homogenization in the connected lake and river ecosystem. This homogeneity of algae compositions was also observed in Dianchi Lake and its downstream river (Yu et al., 2015).

There were significant differences in the phytoplankton community structures and dominant taxa at Dianchi Lake and the three inflow rivers. Previous studies have showed that different anthropogenic activities brought about different sources of pollution, resulting in variations of biological groups and dominant species (Lakhloufi et al., 2021; Pukk et al., 2021; Xie, Zhao, et al., 2021; Xiong et al., 2019). Significant differences in the compositions of phytoplankton community were observed among river sampling sites although there were some common genera such as *Microcystis*, *Cryptomonas*, and *Stephanodiscus*. The predominant taxa in Panlong River included *Chlamydomonas*, *Cocconeis pediculus*, *Stephanodiscus*, *Cryptomonas*, and others, whereas *Uroglena*, *Cryptomonas*, *Acrispumella msimbaziensis* were found in Baoxiang River, and *Conticribra weissflogiopsis*, *Navicula tripunctata*, *Cryptomonas* were found in Chai River. These differences in the compositions of phytoplankton community in the three inflow rivers might be also closely related to different watershed environment and the different sources of pollution. The phytoplankton compositions of Dianchi Lake were partially consistent with that of previous studies, including the dominant cyanobacteria taxon (*Microcystis*) and the dominant eukaryotic phytoplankton taxa (*Stephanodiscus*, *Ceratium* sp. and *Cryptomonas* among others; Feng et al., 2020; Wang et al., 2016; Zhang et al., 2021; Zhang, Zuo, et al., 2020). And most of the predominant phytoplankton taxa (e.g., *Microcystis*, *Navicula* and *Cyclotella*) in the inflow rivers were the same as the historical monitoring results, but there were still some differences. For example, Li, Yang, et al. (2014) found that phytoplankton taxa with high frequency in Chai River, Baoxiang River, and Panlong River also included *Synedra*, *Scenedesmus*, *Ankistrodesmus*, and others; Jin et al. (2018) revealed that the predominant species in Baoxiang River were *Cyclotella meneghiniana*, *Melosira granulata*, *Scenedesmus quadricauda* and others. These differences could be related to the difference in sampling time or the improvement of river water quality caused by comprehensive river management in recent years (Wei, 2016; Yang et al., 2022). Actually, it also could not be ruled out the deviations caused by eDNA metabarcoding technology defectives such as low amplification efficiency and primer bias (Lin & Zhao, 2021); thus, it is necessary to simultaneously conduct morphological monitoring in future actual survey.

### 4.2 Phytoplankton diversities were shaped by different environmental variables which associated with anthropogenic activities in Dianchi Lake and its primary inflow rivers

Uncovering the environmental variables affecting phytoplankton diversity plays key roles to explore the impacts of anthropogenic activities on phytoplankton diversity. Subjecting to various anthropogenic activities such as urbanization, water diversion, and industrial and agricultural activities, lakes and rivers receive a large amount of chemicals, organic pollutants, and nutrients, which results in the changes of environmental variables such as the accumulation of total phosphorus, nitrogen, carbon, and the water eutrophication. These affect the phytoplankton community and diversity rapidly and seriously and even cause blooms of blue green algae (Jin et al., 2017; Li et al., 2022; Yang et al., 2016).

Changes in the Shannon Wiener indices reflected the spatial distribution patterns of phytoplankton diversity among Dianchi Lake and the inflow river sampling sites, which were related to the accumulation of pollutants and nutrients from various anthropogenic disturbances. For example, midstream of Panlong River where suffers from dense population and urban sewage (Yang et al., 2020), and downstream of Chai River where accumulates of abundant N and P from farmland runoff and P mines (Zhang, 2016) were places where the Shannon–Wiener indices went down. The highest diversity index was found in the loci of Panlong Waterfall Park (P3), where Niulan River water was artificially introduced. The inflow of Niulan River water not only increases the runoff of Panlong River and improves the water quality (Mao et al., 2017; Zhang & Wu, 2020), but could also bring more biological groups and enrich the biodiversity of Panlong River and Dianchi Lake.

Environmental variables play important roles in the composition and abundance of the phytoplankton community. Higher temperature and nutrient concentrations facilitate the growth of Cyanobacteria, changing patterns of phytoplankton community (Huo et al., 2022; Li et al., 2020). COD, and pH among others also significantly impact on the growth of dominant phytoplankton taxa and phytoplankton community structure (Zhang, Xu, et al., 2020). In our results, WT positively correlated with the abundances of *Microcystis* and *Dolichospermum*, whereas negatively correlated with *Chlamydomonas*. This was owing to the cooler weather at the time of sampling, and WT became an important limiting factor for phytoplankton growth. Otherwise, TN, COD, pH, and C were closely related to the distribution of dominant genera such as *Acrispumella*, *Uroglena*, and *Cyclotella*, which could lead to the variation of phytoplankton community structures and dominant taxa at different habitats.

Phytoplankton community structures in Dianchi Lake and the inflow rivers were influenced by environmental variables. Due to the strong disturbances of anthropogenic activities, Dianchi Lake has become an eutrophic lake which harmful algal blooms occurred frequently (Wang et al., 2019). C, DO, TN, TP, and COD were identified as primary environmental variables affecting the distribution characteristics of phytoplankton community in Dianchi Lake (Wang et al., 2016). pH, TP, and ${\mathrm{NH}}_{4}^{+}$ positively correlated with the density and biomass of *Microcystis* sp. in Dianchi Lake, whereas ${\mathrm{NO}}_{3}^{-}$, N:P, TN, dissolved organic carbon (DOC) and total organic carbon (TOC) negatively correlated (Feng et al., 2020). This study showed that the diversity patterns of Dianchi Lake sites were relatively homogeneous and closely related to the current pollution situation caused by continuous anthropogenic activities, COD, WT, TP, and pH positively correlated with the phytoplankton community, whereas DO, ${\mathrm{NH}}_{4}^{+}$, TN, and C were negative factors. In the three inflow rivers, distinct biodiversity patterns were observed, which were also closely associated with the impacts of various anthropogenic activities on environmental variables around the sampled sites. Panlong River flows through the primary urban area of Kunming city, dense population along the riverbank brings serious and diverse pollutants into the river, and the inflow of Niulan River water with high turbidity also seriously affects its water quality (Yang et al., 2020; Zhang & Wu, 2020). Thus, the concentrations of TP, COD, and TN in the water were high, the phytoplankton communities in Panlong River were primarily positively related to DO but negatively related to TP and COD. Pollution in the Chai River was primarily from farmland runoff and the export of P from mines, while combined pollution from agricultural and urban non‐point sources occurred in Baoxiang River (Li, Yang, et al., 2014; Zhang, 2016). Thus, the environmental variables correlated with the phytoplankton community structures in both the Baoxiang River and Chai River included TN, C, COD, WT, and TP. And the effects of TP on the communities were discrepant at different loci in Chai River, which were consistent with the output of P in the mines around the Chai River basin (Kong et al., 2012).

All in all, the distribution characteristics of phytoplankton were closely associated with the variations of environmental variables induced by anthropogenic activities, and primary environmental stressors for shaping phytoplankton communities were diagnosed for improving healthy management of Dianchi Lake and its primary inflow rivers. Nutrients (TN and TP) and organic pollutants (COD) were primary environmental stressors, and the effects of these environmental stressors varied in different habitats: TP and COD positively correlated with the phytoplankton communities in Dianchi Lake, whereas TN negatively correlated. TN positively correlated with the phytoplankton communities in Baoxiang River and Chai River, whereas TP and COD negatively correlated with the phytoplankton communities in Panlong River and Baoxiang River. Therefore, eutrophication attributed to the anthropogenic activities could possibly still be a major problem in Dianchi Basin, but unique habitat‐specific governmental measures should be implemented.

## 5 CONCLUSIONS

Based on eDNA metabarcoding, 9 phyla, 30 classes, 84 orders, 132 families, and 243 genera/species phytoplankton taxa were detected in Dianchi Lake and its three inflow rivers. Phytoplankton diversity (e.g., community structure, unique dominant taxon, ɑ‐diversity) showed substantial variability in Dianchi Lake and its three inflow rivers. The relative abundances of dominant taxon and community structure were significantly related to the environmental variables (WT, DO, COD, TP, TN, pH, and C), which were affected by various anthropogenic activities. The primary environmental stressors varied in different habitats, which TP and COD positively correlated with the phytoplankton community in Dianchi Lake whereas negatively correlated in Panlong River and Baoxiang River. TN positively correlated with the phytoplankton community in Baoxiang River and Chai River but negatively correlated in Dianchi Lake. This study provides insights on phytoplankton diversity monitoring and biodiversity conservation and the sustainable management of Dianchi Lake and its three inflow rivers.

## AUTHOR CONTRIBUTIONS

**Yuanyuan Lin:** Formal analysis (lead); funding acquisition (equal); methodology (lead); software (lead); validation (lead); writing – original draft (lead); writing – review and editing (equal). **Wenjun Zhong:** Formal analysis (equal); investigation (lead); methodology (equal). **Xiaowei Zhang:** Conceptualization (equal); supervision (equal); validation (equal). **Xiaohua Zhou:** Conceptualization (equal); investigation (equal). **Liwei He:** Investigation (equal); visualization (equal). **Jiacheng Lv:** Formal analysis (equal); investigation (equal). **Zheng Zhao:** Conceptualization (lead); funding acquisition (lead); supervision (lead).

## CONFLICT OF INTEREST STATEMENT

The authors declare that they have no conflict of interest.

## Supporting information


Figure S1

Table S1

Table S2

Table S3

## Data Availability

Raw sequencing data were deposited in the NCBI Bioproject database (accession number: PRJNA914056).
