# Supplementary figures and images for: Environmental DNA metabarcoding revealed the impacts of anthropogenic activities on phytoplankton diversity in Dianchi Lake and its three inflow rivers
Source: Ecol Evol. 2023 May 19;13(5):e10088. doi: 10.1002/ece3.10088 (PMC10196938; doi:10.1002/ece3.10088)

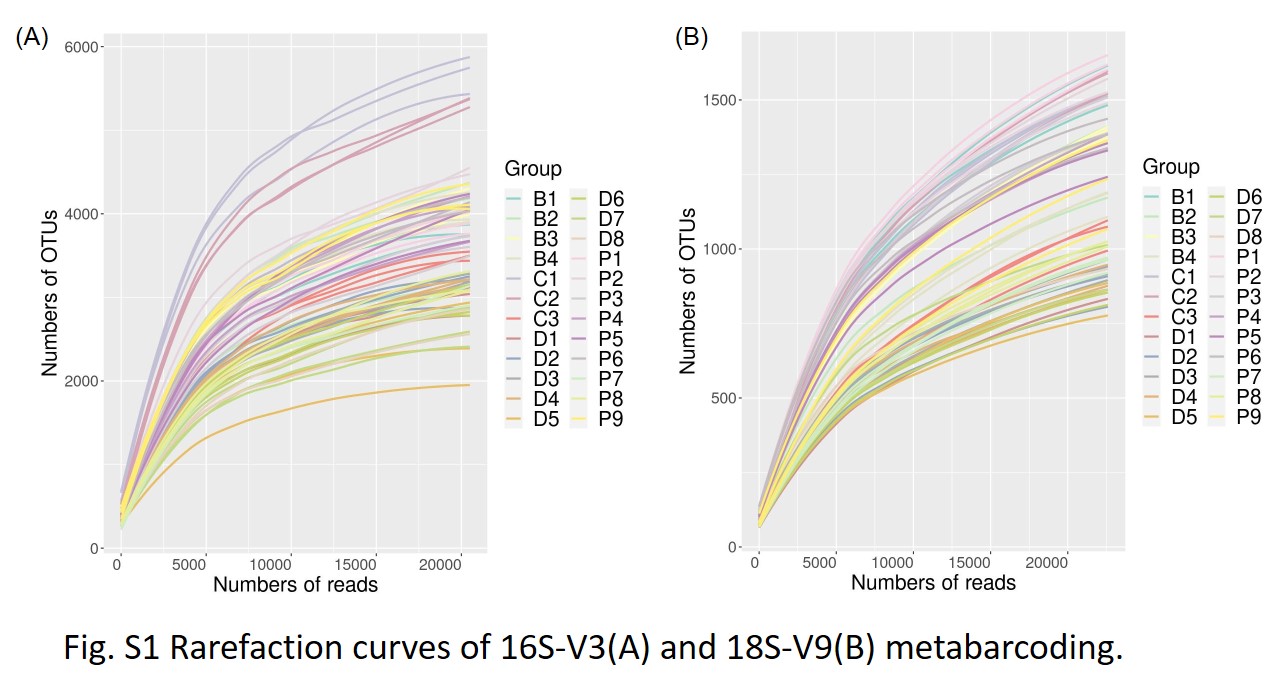

Supplement: Supplementary file 1 — Figure S1 [file ECE3-13-e10088-s002.jpg]
